# Comparative evaluation of weaning and feeding strategies in lamb production: effects on carcass and meat characteristics

**DOI:** 10.1007/s11250-026-04872-7

**Published:** 2026-01-23

**Authors:** Zeki Şahinler, Ömer Faruk Güngör, Yücel Demir

**Affiliations:** 1 Department of Vegetable and Animal Production, Bolu Abant Izzet Baysal University, 14800 Bolu, Türkiye; 2 Department of Veterinary, Bolu Abant Izzet Baysal University, 14800 Bolu, Türkiye; 3 Department of Animal Production and Technologies, Agrı Ibrahim Cecen University, Agri, Türkiye

**Keywords:** Suckling, Grazing, Mutton, Meat quality, Slaughter traits, Carcass characteristics

## Abstract

This study aimed to evaluate the effects of weaning age and three feeding systems on slaughter traits, carcass characteristics, and meat quality in lambs. Forty-five lambs were divided into three groups. Group I lambs were grazed and suckled, Group II lambs were weaned and raised in stable, and Group III lambs were weaned and grazed. Lambs were slaughtered on the 135th day. Body weights on day 75 were similar across groups, but slaughter traits on day 135 were lower in Group III. Head, feet, and heart percentages were significantly higher (*P* < 0.001) in Group I. Additionally, carcass and hind limb compactness indices were significantly higher in Group I than in Group III (*P* = 0.001 and *P* = 0.022, respectively). Group I also had a significantly higher lean percentage (*P* < 0.001), whereas Group II had a significantly higher fat percentage (*P* = 0.008). Carcasses from Groups I and III exhibited greater lightness (*P* = 0.001), and meat from Groups I and II had higher Warner-Bratzler shear force (WBSF) values (*P* = 0.024). In conclusion, Group III exhibited superior meat quality, Group II showed better carcass traits, while Group I demonstrated the most balanced overall performance.

## Introduction

Meat is an important animal protein in the human diet due to its great palatability and rich nutrients. Although the production of lamb meat is an important agricultural activity in the world, the impact of livestock species on meat production varies according to the characteristics of countries. For example, sheep husbandry is more common in Asia, Africa, Australia, New Zealand, and Europe (UK, Spain, Romania, and Greece), where sheep breeds have been reared for meat, milk, and other economic traits according to their geographical, climatic, and socio-economic conditions. In these regions, sheep are typically raised in free-range systems based on natural resources, and mutton is often preferred due to its distinctive flavor and aroma (Cadavez et al. 2020; Güngör et al. 2022, 2023; Yousefi et al. 2019).

Lambs are slaughtered at approximately 6 months of age to ensure quality mutton production because increasing age results in decreased meat quality and yield due to decreased weight gain and tenderness (Prache et al. 2022). After weaning, lambs are typically fattened either in stable or on pasture until slaughter. However, weaning is a stressful transition period for suckling lambs that can significantly affect meat production. Consequently, keeping lambs unweaned on pasture until slaughter is a common practice in Türkiye. Feeding (intensive vs. grazing) and weaning (early vs. normal) strategies for lambs can be categorized into two main approaches. In intensive feeding systems, the lambs are fed with concentrated feed and some green fodder. Grazing on the pasture, which is quite usual in the steppe climate regions, the lambs are reared with pasture. The weaning management of the lambs can be divided into two main methods. Early weaning of lambs is generally preferred method for milk-producing farms, but traditionally reared lambs are usually weaned at 2–3 months of age (Ma et al. 2021; McCoard et al. 2020; Simeonov and Harmon 2021). However, the stress of weaning can reduce slaughter performance and meat quality, so the evaluation cost-effective feed management systems is important.

It is known that management and feeding systems affect growth, development, slaughter, and meat characteristics. Therefore, determination of the best way according to the farm conditions is important because the sheep farmer is mainly a lamb producer, where the carcass traits have great importance. Their main objective is to produce lambs of a certain weight at a certain age at the end of the targeted fattening period, considering economically important carcass traits. Additionally, these traits are also important for the production, growth, and development of breeding stock. Live weight expresses meat yield and is therefore the most important performance and economic trait. However, it is not descriptive without slaughter and carcass traits such as carcass components and composition (Altınel et al. 2001; Delgado-Pando et al. 2021; Ellies-Oury et al. 2020). In addition, the type of feeding system and the weaning age of lambs are important for meat quality.

The age of weaning and the method of feeding are known to influence not only growth and development but also the carcass traits and meat quality traits of lambs. Therefore, the aim of this study is to assess the effects of weaning age and feeding system (intensive in stable or grazing on the land) on slaughter traits and meat quality of lambs.

## Materials and methods

Animal procedures in this study were conducted as part of routine livestock practices in the Agri Province region of Türkiye. No experimental interventions were performed beyond standard husbandry operations. According to the Regulation on the Welfare and Protection of Animals Used for Experimental and Other Scientific Purposes “This Regulation does not cover non-experimental agricultural and clinical veterinary practices.” Therefore, the study is exempt from Local Ethics Committee approval and is not subject to permission from the Republic of Türkiye Ministry of Agriculture and Forestry. This study was supported by the Republic of Türkiye Ministry of Agriculture and Forestry, General Directorate of Agricultural Research and Policies (TAGEM), as part (No: 04MOR2013-1) of the “National Genetic Improvement Project for Small Ruminants Under Breeder Conditions”.

### Geographical location, animal material, and feeding management

This study was performed in the central province of Ağrı (39°56’44.6"N 43°08’56.5” E) in the East Anatolian Region of Türkiye. Forty-five male lambs (3 groups x 15 lambs) born singleton were used in this trial. The lambs used in this study were of the Morkaraman (Red Karaman) breed, an indigenous fat-tailed sheep from East Anatolia, well adapted to the region’s climate and grazing conditions (Akçapınar 2000). Lambs in the groups were kept together with their dams until the 75th day (weaning time). The lambs were divided into 3 groups after weaning and reared in these groups consisting of 15 lambs until slaughter. However, one of the groups was not weaned. One of the two weaned groups was reared indoors with concentrated feed, while the other was reared outdoors on pasture.

Based on data obtained from the Ağrı Provincial Directorate of Agriculture, the vegetation cover of pasturelands in the region is structured as follows: approximately 20% comprises *Medicago sp.*,* Onobrychis sativa*,* Dactylis glomerata*,* Agropyron sp.*,* Bromus tomentellus*,* Lotus corniculatus*,* Koeleria cristata*,* Trifolium sp.*,* Poa sp.*,* Agropyron tricofon*, and *Phleum montana*. About 40% consist of *Festuca ovina*,* Astragalus sp.*,* Thymus parviflora*,* Plantago major*,* Coronilla orientalis*,* Cynodon dactylon*,* Carex sp*., and *Fescuta sp*. The remaining 40% include *Salvia officinalis*,* Falcaria vulgaris*,* Achillea millefolium*,* Anthemis sp.*,* Hypericum perforatum*,* Cephalaria syriaca*,* Helichrysum sp.*,* Eryngium campestre*,* Galium verum*,* Verbascum sp*., and *Taraxacum officinale*.

All animals were provided with potable water three times a day. The lambs in each group were of similar age, as they were randomly selected from a flock of 300 ewes during the three peak lambing days. The 75th live weights of groups I, II, and III were 21.27 ± 0.82, 20.65 ± 0.57, and 20.57 ± 0.83 kg (*P* = 0.771), respectively.

Group I: The group of suckling outdoor lamb was reared with grazing on pasture and ad libitum sucking every morning. The dams of lambs were fed only with pasture grazing.

Group II: The group of the weaned indoor lambs was reared with ad libitum lamb grower feed (16% crude protein, 2500 kcal ME/kg energy, 9.5% crude cellulose, 6% crude ash) + hay-clover after the 75th day of age.

Group III: The group of the weaned outdoor lambs was reared only with ad libitum grazing on pasture after the 75th day.

### Slaughtering and carcass characteristics

All animals at the age of 135th day were weighed on an electronic scale and slaughtered after 12 h fasting period. The animals were slaughtered without stunning by the halal procedure, according to the routine commercial practice in Türkiye. Non-carcass components listed in Table 1, except kidneys and perinephric-pelvic fat, were removed after bleeding, and all these components and the hot carcass were weighed and recorded. Subsequently, the carcass measurements and conformation indicators (Table 2) were determined in accordance with the reports of Ekiz et al. (2010), Cañeque et al. (2004), Fisher and de Boer (1994), and Santos et al. (2007). The cold carcass weight was identified after the carcass was kept at 4 ^◦^C for 24 h after slaughter.

After the tail fat, kidneys, and perinephric-pelvic fat removing and weighed, the cold carcasses were longitudinally cut along the vertebral column, and these two half carcasses were weighed. The right halves of the carcasses were cut into 5 parts (shoulder, neck, flank, ribs, and hind limb) according to Colomer-Rocher et al. (1987), and the percentage of carcass parts was calculated based on cold carcass weight. For the determination of the carcass composition, these parts were dissected into lean, bone, fat, and remainder (Fisher and de Boer 1994). Rib eye areas (cm^2^) were measured between the 12th and 13th ribs on the LTL muscle section (Boggs and Merkel 1993).

### Meat quality traits

Muscle pH was measured in LTL muscle at 0 and 24 h postmortem using a digital pH meter. (Testo 205, Testo AG, Lenzkirch, Germany). The pH meter was calibrated at chiller temperatures 30 min before the first measurement of the carcass pH. The Warner-Bratzler Shear Force (WBSF) values were measured on six subsamples (1 × 1 cm thick, 2.5–3 cm length) taken from the LTL muscle using an Instron testing device (Model 3343, Instron Corp., Norwood, MA, USA). Meat color traits (L*: lightness, a*: redness, b*: yellowness) were determined at 4 ◦C on the cut surface of 3 cm thick LTL samples using a Minolta chromometer (Minolta CR 400, Minolta Camera Co., Osaka, Japan) at the 0-, 1- and 24-hours postmortem. The light source, aperture size, and observation angle were set as illuminant D65, 8 mm, and 2^◦^, respectively. Chroma (C *) and hue angle (H *) were calculated according to the formula of Wyszecki and Stiles (1982).

### Statistical analysis

All the results of the slaughter traits and carcass characteristics were analyzed using the general linear model procedure with LSD in a model adjusted by analysis of covariance for differences in the appropriate covariate (this information was given under the tables). The one-way analysis of variance (ANOVA) with Tukey’s HSD test (post hoc) was used to calculate the live weights comparison to the meat quality results. All statistical analysis was performed with SPSS version 22 (SPSS 2013).

## Results

### Slaughter traits and non-carcass components

The slaughter traits and carcass component results were given in Table 1. The results indicate that all carcass traits, except for cooler shrinkage, were influenced by weaning age and feeding methods. The evaluation of non-carcass component percentages showed that these differences had a significant effect only on the head, feet, heart, empty compartment, and content of the digestive tract values.

**Table 1:** Slaughter traits and non-carcass components (Mean ± SE)

Items	Group I	Group II	Group III	*P* values	Cov.
Slaughter traits					
Slaughter weight (kg)	33.38 ± 0.89^a^	35.33 ± 0.96^a^	27.54 ± 0.97^b^	**< 0.001**	0.123
Empty body weight (kg)	29.77 ± 0.75^a^	30.26 ± 0.81^a^	23.69 ± 0.82^b^	**< 0.001**	0.088
Hot carcass weight (kg)	15.39 ± 0.16^a^	15.17 ± 0.19^a^	14.31 ± 0.21^b^	**0.002**	0.000
Hot dressing^A^ (%)	47.82 ± 0.51^a^	47.12 ± 0.59^a^	44.40 ± 0.65^b^	**0.002**	0.839
Hot dressing^B^ (%)	53.54 ± 0.40^a^	54.65 ± 0.47^a^	51.98 ± 0.52^b^	**0.008**	0.186
Cold carcass weight (kg)	14.92 ± 0.18^a^	14.78 ± 0.21^a^	13.82 ± 0.23^b^	**0.004**	0.000
Chilled dressing^A^ (%)	46.31 ± 0.54^a^	45.82 ± 0.63^a^	42.85 ± 0.70^b^	**0.003**	0.442
Chilled dressing^B^ (%)	51.85 ± 0.46^a^	53.15 ± 0.53^a^	50.15 ± 0.59^b^	**0.009**	0.059
Cooler shrink	3.19 ± 0.31	2.76 ± 0.36	3.55 ± 0.40	0.437	0.034
Non-carcass components (as % of SW)					
Blood	8.92 ± 0.25	8.32 ± 0.29	8.73 ± 0.32	0.257	0.883
Head	6.28 ± 0.07^a^	5.80 ± 0.08^b^	5.99 ± 0.09^b^	**< 0.001**	0.000
Feet	2.51 ± 0.04^a^	2.17 ± 0.05^b^	2.48 ± 0.05^a^	**< 0.001**	0.003
Pelt	9.79 ± 0.27	9.36 ± 0.31	10.37 ± 0.35	0.186	0.107
Pluck unit	4.75 ± 0.08^a^	4.36 ± 0.10^b^	4.61 ± 0.11^ab^	**0.008**	0.009
Lungs and trachea	1.37 ± 0.04	1.33 ± 0.05	1.31 ± 0.05	0.599	0.002
Heart	0.47 ± 0.01^a^	0.39 ± 0.01^b^	0.41 ± 0.01^b^	**< 0.001**	0.000
Liver	2.08 ± 0.07	1.87 ± 0.08	2.04 ± 0.09	0.134	0.223
Spleen	0.28 ± 0.03	0.20 ± 0.04	0.27 ± 0.04	0.236	0.167
Pluck remainder	0.56 ± 0.03	0.56 ± 0.04	0.57 ± 0.04	0.963	0.517
Empty digestive tract	8.46 ± 0.22	8.31 ± 0.26	8.23 ± 0.29	0.795	0.040
Compartments	4.04 ± 0.15^a^	3.44 ± 0.17^b^	3.80 ± 0.19^ab^	**0.030**	0.579
Intestine	4.42 ± 0.17	4.87 ± 0.20	4.43 ± 0.23	0.208	0.030
Contents of the digestive tract	10.70 ± 0.50^b^	13.74 ± 0.58^a^	14.62 ± 0.64^a^	**< 0.001**	0.162
Compartments	7.10 ± 0.47^b^	8.77 ± 0.54^a^	9.83 ± 0.61^a^	**0.002**	0.398
Intestine	3.61 ± 0.27^b^	4.98 ± 0.31^a^	4.79 ± 0.35^a^	**0.001**	0.261
Omental fat	0.35 ± 0.04	0.40 ± 0.04	0.27 ± 0.05	0.266	0.590
Testicles	0.42 ± 0.03	0.43 ± 0.04	0.32 ± 0.04	0.231	0.665

A: Based on slaughter weight, B: Based on empty body weight, Cov.: The covariate was slaughter weight for all variables except for slaughter weight and empty body weight the covariate was birth weight, SE: Standard error; a, b: Means with unlike letters in rows differ significantly (*P* < 0.05)

### Carcass measurements

When the carcass measurements were examined, the evaluated factors had a significant effect on all carcass measurements but chest measurements, and internal carcass and leg lengths (Table 2).

**Table 2:** Carcass measurements (Mean ± SE)

Items	Group I	Group II	Group III	*P* values	Cov.
Lengths (cm)					
Carcass	64.20 ± 0.61^a^	61.92 ± 0.71^b^	65.24 ± 0.79^a^	**0.017**	0.001
Internal carcass	59.00 ± 0.37	58.65 ± 0.43	59.71 ± 0.49	0.383	0.000
Back	54.42 ± 0.53^a^	52.47 ± 0.62^b^	56.11 ± 0.69^a^	**0.005**	0.000
Leg external	35.21 ± 0.30^a^	34.12 ± 0.34^b^	35.57 ± 0.38^a^	**0.025**	0.000
Leg internal	17.00 ± 0.76	17.73 ± 0.89	16.67 ± 0.99	0.742	0.999
Widths (cm)					
Chest	17.01 ± 0.31	17.83 ± 0.36	17.17 ± 0.40	0.195	0.000
Rump	17.49 ± 0.36^a^	15.72 ± 0.41^b^	16.12 ± 0.46^b^	**0.002**	0.004
Depths (cm)					
Chest	27.36 ± 0.17	27.13 ± 0.20	27.25 ± 0.22	0.633	0.000
Internal chest	18.01 ± 0.19^a^	16.70 ± 0.22^b^	16.42 ± 0.24^b^	**< 0.001**	0.032
Girths (cm)					
Chest	71.62 ± 0.51	71.54 ± 0.59	70.24 ± 0.66	0.305	0.000
Rump	52.38 ± 0.49^a^	50.51 ± 0.57^b^	52.24 ± 0.64^ab^	**0.038**	0.000
Indices					
Carcass compactness g/cm	252.33 ± 3.17^a^	250.98 ± 3.68^a^	230.47 ± 4.11^b^	**0.001**	0.000
Hind limb compactness, g/cm	63.03 ± 0.95^a^	60.46 ± 1.10^ab^	58.79 ± 1.23^b^	**0.022**	0.000
Chest roundness	0.62 ± 0.01	0.66 ± 0.01	0.63 ± 0.01	0.084	0.004

Cov.: The covariate was slaughter weight; SE: Standard error; a, b: Means with unlike letters in rows differ significantly (*P* < 0.05); Carcass compactness: hot carcass weight/internal carcass length; Hind limb compactness: leg weight/hind limb length; Chest roundness: carcass depth/thoracic depth

### Carcass components and composition

Individual carcass parts were significantly different among the groups except neck, rib, and tail. The composition of the carcass was significantly influenced by feeding management, except for bone%. Additionally, the lean/fat, lean/bone, and backfat thickness values were also affected by feeding managements except rib eye area (Table 3).

**Table 3:** Components and composition of the carcass (Mean ± SE)

Items	Group I	Group II	Group III	*P* values	Cov.
Components in the left half carcass (out of 100)					
Shoulder	16.90 ± 0.21^b^	16.37 ± 0.24^b^	17.77 ± 0.29^a^	**0.012**	0.001
Flank	9.42 ± 0.24^b^	9.61 ± 0.27^b^	10.76 ± 0.33^a^	**0.017**	0.053
Neck	7.62 ± 0.23	7.71 ± 0.26	7.39 ± 0.31	0.803	0.526
Ribs	18.40 ± 0.48	18.14 ± 0.55	16.74 ± 0.66	0.201	0.196
Anterior rib	12.24 ± 0.34	11.43 ± 0.39	10.89 ± 0.46	0.053	0.201
Loin rib	6.16 ± 0.26	6.71 ± 0.30	5.85 ± 0.36	0.217	0.462
Hind Limb	30.46 ± 0.41^a^	28.53 ± 0.48^b^	31.22 ± 0.57^a^	**0.002**	0.056
Others					
Tail	15.75 ± 0.75	17.87 ± 0.86	14.89 ± 1.03	0.086	0.009
Kidneys	1.14 ± 0.05^b^	1.28 ± 0.06^a^	0.99 ± 0.07^b^	**0.021**	0.005
Perinephric–pelvic fat	0.31 ± 0.04^b^	0.50 ± 0.04^a^	0.25 ± 0.05^b^	**0.001**	0.411
Composition of the carcass (out of 100)					
Lean	64.29 ± 0.56^a^	60.66 ± 0.64^b^	61.90 ± 0.76^b^	**< 0.001**	0.331
Bone	22.81 ± 0.41	23.12 ± 0.47	23.64 ± 0.55	0.525	0.001
Fat	9.55 ± 0.58^b^	12.20 ± 0.67^a^	9.96 ± 0.79^ab^	**0.008**	0.001
Remainder	3.36 ± 0.24^b^	4.03 ± 0.27^a^	4.50 ± 0.32^a^	**0.016**	0.895
Lean/Fat	7.48 ± 0.53^a^	5.45 ± 0.61^b^	7.00 ± 0.72^ab^	**0.028**	0.012
Lean/Bone	2.84 ± 0.06^a^	2.65 ± 0.07^b^	2.64 ± 0.08^b^	**0.024**	0.015
Backfat thickness, cm	1.07 ± 0.12^b^	1.53 ± 0.14^a^	0.87 ± 0.17^b^	**0.013**	0.014
Rib eye area (cm^2^)	10.10 ± 0.39	8.97 ± 0.45	9.94 ± 0.54	0.127	0.000

Cov.: The covariate was cold carcass weight; SE: Standard error; a, b: Means with unlike letters in rows differ significantly (*P* < 0.05)

### Composition of the carcass components

Lean percentages of the carcass parts were significantly changed among the groups, except for ribs. With the exception of the hind limb, the percentage of bone in these parts did not differ. The fat percentage of the shoulder, flank and neck, and the remainder percentage of neck and hind limb influenced from the factors; however, the fat and remainder percentages of the other parts did not influence from the factors (Table 4).

**Table 4:** Composition of the carcass cuts (Mean ± SE)

Items	Group I	Group II	Group III	*P* values	Cov.
Shoulder					
Lean	66.60 ± 0.73^a^	62.22 ± 0.84^b^	63.93 ± 0.99^ab^	**< 0.001**	0.297
Bone	21.72 ± 0.47	22.05 ± 0.53	22.73 ± 0.64	0.493	0.101
Fat	8.33 ± 0.68^b^	12.05 ± 0.78^a^	9.65 ± 0.93^ab^	**0.001**	0.018
Remainder	3.35 ± 0.33	3.68 ± 0.38	3.70 ± 0.45	0.692	0.709
Flank					
Lean	56.03 ± 1.17^a^	50.73 ± 1.35^b^	55.37 ± 1.60^ab^	**0.008**	0.226
Bone	19.98 ± 0.82	19.64 ± 0.94	20.09 ± 1.12	0.949	0.277
Fat	20.05 ± 1.16^b^	26.59 ± 1.33^a^	20.82 ± 1.58^b^	**0.001**	0.038
Remainder	3.95 ± 0.42	3.03 ± 0.48	3.73 ± 0.57	0.298	0.777
Neck					
Lean	66.81 ± 0.84^a^	62.44 ± 0.96^b^	63.71 ± 1.14^ab^	**0.001**	0.718
Bone	22.74 ± 0.88	22.13 ± 1.01	20.03 ± 1.20	0.268	0.091
Fat	4.74 ± 0.64^b^	7.87 ± 0.73^a^	7.62 ± 0.87^a^	**0.001**	0.121
Remainder	5.71 ± 0.58^b^	7.56 ± 0.67^a^	8.64 ± 0.79^a^	**0.009**	0.706
Ribs					
Lean	59.23 ± 1.12	56.43 ± 1.29	55.32 ± 1.53	0.069	0.766
Bone	29.78 ± 1.40	28.47 ± 1.61	31.93 ± 1.91	0.508	0.045
Fat	7.12 ± 1.22	9.57 ± 1.40	6.22 ± 1.66	0.284	0.012
Remainder	3.87 ± 1.10	5.53 ± 1.26	6.52 ± 1.50	0.308	0.990
Hind Limb					
Lean	67.20 ± 0.63^a^	64.70 ± 0.73^b^	65.21 ± 0.86^ab^	**0.013**	0.698
Bone	21.39 ± 0.47^b^	22.81 ± 0.53^a^	23.26 ± 0.63^a^	**0.025**	0.002
Fat	9.12 ± 0.57	9.55 ± 0.65	8.17 ± 0.78	0.524	0.004
Remainder	2.30 ± 0.19^b^	2.94 ± 0.22^a^	3.36 ± 0.26^a^	**0.004**	0.935

Cov.: The covariate was cold carcass weight, SE: Standard error; a, b, c: Means with unlike letters in rows differ significantly (*P* < 0.05)

### Meat quality characteristics

Meat quality traits are shown in Table 5. The WBSF and meat color values were generally affected by evaluated factors, but the pH values were not. When the color traits were analyzed, most color values were generally found to have a significant difference among the groups, even though the differences decreased after the 1st hour after slaughter.

**Table 5:** Meat quality traits (Mean ± SE)

Items	Group I	Group II	Group III	*P* values
pH				
0th hour	6.50 ± 0.05	6.57 ± 0.03	6.42 ± 0.06	0.072
24th hour	5.70 ± 0.10	5.52 ± 0.07	5.50 ± 0.05	0.141
0–24 h	0.80 ± 0.10	1.05 ± 0.08	0.92 ± 0.08	0.129
WBSF	2.94 ± 0.14^a^	3.20 ± 0.17^a^	2.37 ± 0.13^b^	**0.001**
Meat color traits				
0th hour				
Lightness	38.91 ± 0.59^a^	37.77 ± 0.59^ab^	39.86 ± 0.50^b^	**0.039**
Redness	16.02 ± 0.22	15.65 ± 0.16	16.34 ± 0.21	0.064
Yellowness	0.51 ± 0.29^b^	-0.40 ± 0.24^a^	1.11 ± 0.21^b^	**0.001**
Chroma	16.06 ± 0.22^a^	15.68 ± 0.16^ab^	16.39 ± 0.21^b^	**0.051**
Hue	1.74 ± 1.04^b^	-1.49 ± 0.89^a^	3.92 ± 0.76^b^	**0.001**
1st hour				
Lightness	40.10 ± 0.45^b^	38.51 ± 0.42^a^	40.55 ± 0.44^b^	**0.005**
Redness	18.04 ± 0.31	18.14 ± 0.28	18.16 ± 0.33	0.951
Yellowness	4.98 ± 0.34	4.75 ± 0.34	5.91 ± 0.42	0.078
Chroma	18.74 ± 0.36	18.78 ± 0.34	19.15 ± 0.39	0.676
Hue	15.30 ± 0.90^ab^	14.52 ± 0.86^a^	17.86 ± 1.15^b^	0.051
24th hour				
Lightness	41.12 ± 0.42^b^	39.69 ± 0.41^a^	41.17 ± 0.43^b^	**0.024**
Redness	16.99 ± 0.37^a^	18.50 ± 0.29^b^	17.84 ± 0.33^ab^	**0.010**
Yellowness	7.19 ± 0.20	7.02 ± 0.207	7.49 ± 0.18	0.244
Chroma	18.46 ± 0.40^a^	19.79 ± 0.33^b^	19.36 ± 0.33^ab^	**0.033**
Hue	22.96 ± 0.46^b^	20.74 ± 0.41^a^	22.80 ± 0.51^b^	**0.002**

SE: Standard error; a, b: Means with unlike letters in rows differ significantly (*P* < 0.05); WBSF: Warner Bratzler shear force value; L: lightness, a: redness, b: yellowness

## Discussion

In early spring, the lambing season is generally intensified because sheep are usually seasonally polyestrous, and lambs were traditionally reared on grazing resources. For lamb production, pre-slaughter feeding management is therefore divided into 3 types in this study. The first is grazing of un-weaned lambs (Group I), the second is concentrate-based feeding of weaned lambs (Group II), and the third is continued grazing of weaned lambs (Group III). Therefore, these three different types of feeding management were evaluated and compared for their effect on the slaughter traits, carcass characteristics, and meat quality of singleton lambs slaughtered at the same age in this study.

### Slaughter traits and non-carcass components

The groups had a significant effect on all slaughter traits (SW, empty body weight, hot carcass weight, hot dressing, cold carcass weight, and chilled dressing) except for cooler shrink. It was noteworthy that Group I (suckling - grazing) performed better than Group III (weaned - grazing) but were similar with Group II (weaned - in stable). Studies showed that the grazing lambs had lower slaughter performance than concentrated fed lambs (Borton et al. 2005; Carrasco et al. 2009; Diaz et al. 2002; Moron-Fuenmayor et al. 1999; Priolo et al. 2002; Santos-Silva et al. 2002), and the results of this study were in accordance with this information because when comparing the slaughter traits of Group II show higher performance than Group III. Group I, however, had a similar slaughter trait to Group II. This outcome is attributed to carcass composition, as Group I exhibited a higher lean-to-fat ratio compared to Group II (Table 3). This difference is to be expected because grazing leads to higher muscle activity and this leads to lower fat and a higher muscle mass (Carrasco et al. 2009; Cividini et al. 2007). However, the better slaughter performance of suckling-grazing lambs than those of Group III lambs was attributed to the absence of weaning stress and the positive effect of milk on lamb muscle development. Although not significant, the lowest cooler shrinkage value was recorded for the lambs in the concentrate-fed system. This was due to the increased fat content in the carcass (Hanoglu Oral et al. 2023; Joy et al. 2008; Smith et al. 1973). The head and heart percentages of suckling lambs (Group I) were significantly higher than those of weaned lambs (Group II and III). Development can be said to be better in Group I (suckling lambs) because the proportion of these organs was reported to increase with development (Ekiz et al. 2013, 2020). Additionally, this result was consistent with the Cividini et al. (2007). The feet percentages of grazed lambs (Group I and III) were higher than Group II. This is an expected result because the lambs in Group II are less active than the lambs in grazing (Group I and III). The compartments and the digestive tract content percentages were significantly different among the groups. The results were evaluated together, it can be said that suckling and grazing had a positive effect on the digestive system.

### Carcass measurements

In general, there was an increase in the value of the carcass measurements in Groups II, III, and I, respectively. It was understood that the development of pasture-reared lambs was better because of the higher exposure to exercise. This result is similar to the results of Cividini et al. (2007) but different from the results of Ekiz et al. (2020). The carcass and hind limb compactness values were Group I, II, and III in descending order. The slaughter weights of Group I and II were higher than those of Group III. Therefore, the current results on carcass and hind limb compactness were evaluated as expected results, and these results were in accordance with the previous studies (Carrasco et al. 2009; Cividini et al. 2007; Ekiz et al. 2020).

### Carcass components and composition

When the carcass components were evaluated in general, the highest percentages of carcass components with low fat accumulation and high muscle tissue (shoulder and hind limb) were found in groups III, I and II, respectively. These differentiations were generally significant. However, for the carcass components with high fat accumulation (tail and perinephric-pelvic fat), the ranking of the components was in groups II, I, and III. This is a consequence of grazing and is consistent with earlier studies (Ates et al. 2020; Bittante et al. 2021; Ekiz et al. 2020; Hanoglu Oral et al. 2023).

Group I lambs had a higher lean percentage and lean/bone ratio and a lower remainder percentage than the others, while Group II had a higher fat percentage and lower lean/fat ratio than Group I lambs. This shows that suckling and grazing (Group I) until the 135th day is better for muscle development and mutton production. In this study, the high fat percentage on the carcass in stable feeding (Group II) is also reported in many other publications (Ates et al. 2020; Carrasco et al. 2009 Cividini et al. 2007; Priolo et al. 2002).

### Composition of the carcass components

It was reported that the fat and lean percentages vary among the components of the carcass, the fat percentage is lowest in the legs and highest in the flank, the lean percentage is opposite to the fat percentage (Campo et al. 2016; Diaz et al. 2006; Güngör et al. 2022;). The composition of the carcass components obtained in this study is consistent with these findings. Although the differences among the composition values of the ribs were not significant among the groups, when the composition of the carcass components was evaluated in general, the different results among the groups were consistent with the general results of the carcass composition.

### Meat quality characteristics

The pH results are reported to be unaffected by the feeding system (Bittante et al. 2021; dos Santos et al. 2024; Hanoglu Oral et al. 2023; Yilmaz et al. 2023; Priolo et al. 2002;), with exceptions (Cividini et al. 2020; Hopkins et al. 1998). In this study, the ultimate pH (pH 24) differences among the groups were not significant, although the post-slaughter (0 h) pH differences were close to the significant level. Sheep studies report that the ultimate pH should be between 5.50 and 5.70 (Güngör et al. 2020, 2023; Hopkins and Mortimer 2014; Miranda-de la Lama et al. 2012), and the ultimate pH values obtained in this study were 5.70, 5.52, and 5.50 for groups I, II, and III, respectively.

The WBSF value of lamb meat was reported to be 3.00 and above in the studies. The effect of the feeding system on the WBSF was reported in some of these studies, while it was not reported in others (Bittante et al. 2021; dos Santos et al. 2024; Hanoglu Oral et al. 2023; Hopkins et al. 1998; Priolo et al. 2002; Yilmaz et al. 2023; ). It can be said that grazing positively affects WBSF because the WBSF values of groups II were found to be higher than those of Groups III and I. In the consumer panels, the WBSF of more than 5.5 kg is reported to be tough (Shackelford et al. 1991). The WB shear force values in this study were lower than this value, which is a sign that the meat in this study has a soft character.

Meat color differences between groups post-slaughter (0 h) were found to be significant, except for redness, which was close to the significance level. These differences remained significant at 24 h postmortem except for yellowness. The lightness of the carcasses of the grazed lambs (Group I and III) at 24 h postmortem was higher than that of the lambs reared in stable (Group II). There are reports that the lightness value of lamb meat should be 34 and above, and if it is below this value, it has a negative impact on consumer demand (Hopkins et al. 1999). The lightness values observed in this study were considerably higher than 34. In the present study, meat lightness values were higher in lambs grazed on pasture (Groups I and III) compared to those finished indoors (Group II), which contrasts with several previous studies reporting darker meat in pasture-raised lambs (Ekiz et al. 2012, 2019; Joy et al. 2008; Priolo et al. 2002; Yilmaz et al. 2023), but the results of Cividini et al. (2007) are consistent with the study results. These studies attributed the darker meat color to increased physical activity and oxygen demand in grazing lambs, as well as higher ultimate pH and slaughter age. However, in the current study, slaughter age and ultimate pH values were similar across groups, suggesting that the observed differences in lightness may be influenced by other factors such as breed-specific responses, pasture composition, and milk intake in suckling lambs. Additionally, Group II exhibited higher chroma and lower hue values, indicating a more vivid and red-toned meat color, which aligns with findings from concentrate-fed lambs in previous research (Ekiz et al. 2020).

## Conclusion

While Groups I and II showed superior slaughter and carcass traits compared to Group III, Groups I and III exhibited enhanced meat quality relative to Group II. These findings suggest that maintaining lambs under unweaned grazing conditions (Group I) until 135 days of age is an effective strategy for optimizing both carcass and meat quality traits.

## Data Availability

Data will be made available on request.
